# Estrogen receptor mutations and splice variants determined in liquid biopsies from metastatic breast cancer patients

**DOI:** 10.1002/1878-0261.12147

**Published:** 2017-11-17

**Authors:** Nick Beije, Anieta M. Sieuwerts, Jaco Kraan, Ngoc M. Van, Wendy Onstenk, Silvia R. Vitale, Michelle van der Vlugt‐Daane, Luc Y. Dirix, Anja Brouwer, Paul Hamberg, Felix E. de Jongh, Agnes Jager, Caroline M. Seynaeve, Maurice P. H. M. Jansen, John A. Foekens, John W. M. Martens, Stefan Sleijfer

**Affiliations:** ^1^ Erasmus MC Cancer Institute Department of Medical Oncology and Cancer Genomics Netherlands Erasmus University Medical Center Rotterdam The Netherlands; ^2^ Department of Clinical and Molecular Medicine University of Catania Italy; ^3^ Translational Cancer Research Unit Department of Medical Oncology Oncology Center GZA Hospital Sint Augustinus Antwerp Belgium; ^4^ Department of Internal Medicine Franciscus Gasthuis Rotterdam The Netherlands; ^5^ Department of Internal Medicine Ikazia Hospital Rotterdam The Netherlands

**Keywords:** cell‐free DNA, circulating tumor cells, endocrine resistance, ESR1 mutations

## Abstract

Mutations and splice variants in the estrogen receptor (ER) gene, *ESR1*, may yield endocrine resistance in metastatic breast cancer (MBC) patients. These putative endocrine resistance markers are likely to emerge during treatment, and therefore, its detection in liquid biopsies, such as circulating tumor cells (CTCs) and cell‐free DNA (cfDNA), is of great interest. This research aimed to determine whether *ESR1* mutations and splice variants occur more frequently in CTCs of MBC patients progressing on endocrine treatment. In addition, the presence of *ESR1* mutations was evaluated in matched cfDNA and compared to CTCs. CellSearch‐enriched CTC fractions (≥5/7.5 mL) of two MBC cohorts were evaluated, namely (a) patients starting first‐line endocrine therapy (*n* = 43, baseline cohort) and (b) patients progressing on any line of endocrine therapy (*n* = 40, progressing cohort). *ESR1* hotspot mutations (D538G and Y537S/N/C) were evaluated in CTC‐enriched DNA using digital PCR and compared with matched cfDNA (*n* = 18 baseline cohort; *n* = 26 progressing cohort). Expression of *ESR1* full‐length and 4 of its splice variants (∆5, ∆7, 36 kDa, and 46 kDa) was evaluated in CTC‐enriched mRNA. It was observed that in the CTCs, the *ESR1* mutations were not enriched in the progressing cohort (8%), when compared with the baseline cohort (5%) (*P *=* *0.66). In the cfDNA, however, *ESR1* mutations were more prevalent in the progressing cohort (42%) than in the baseline cohort (11%) (*P *=* *0.04). Three of the same mutations were observed in both CTCs and cfDNA, 1 mutation in CTCs only, and 11 in cfDNA only. Only the ∆5 *ESR1* splice variant was CTC‐specific expressed, but was not enriched in the progressing cohort. In conclusion, sensitivity for detecting *ESR1* mutations in CTC‐enriched fractions was lower than for cfDNA. *ESR1* mutations detected in cfDNA, rarely present at the start of first‐line endocrine therapy, were enriched at progression, strongly suggesting a role in conferring endocrine resistance in MBC.

**Abbreviations**
AI: aromatase inhibitor
cfDNA: cell‐free DNA
CTC: circulating tumor cell
dPCR: digital PCR
HBD: healthy blood donor
MBC: metastatic breast cancer
PD: progressive disease
SD: standard deviation
VAF: variant allele frequency

## 1 Introduction

Endocrine therapy is the mainstay of treatment for estrogen receptor (ER)‐positive metastatic breast cancer (MBC) patients. However, 40% of these patients obtain no clinical benefit from first‐line endocrine therapy, and virtually all of the patients in whom the tumor initially responds will eventually develop resistance (Pritchard, 2013). Several mechanisms have been linked to endocrine resistance (De Marchi *et al*., 2016), but none of these have been implemented in daily clinical practice because their clinical value could not be confirmed, or was not strong enough. One recently revealed mechanism for acquired resistance is the emergence of mutations in the gene coding for ER, *ESR1*, yielding a constitutively activated ER. Functional studies have suggested that tumor cells with these mutations are less responsive to estrogen deprivation as induced by aromatase inhibitors (AIs) (Robinson *et al*., 2013; Toy *et al*., 2013), but may still experience growth inhibition by ER‐blocking agents such as tamoxifen and fulvestrant (Jeselsohn *et al*., 2014; Robinson *et al*., 2013; Toy *et al*., 2013). This was recently supported in a retrospective clinical analysis, in which a modest progression‐free survival benefit was observed for MBC patients with an *ESR1* mutation who were treated with fulvestrant, when compared to the AI exemestane (Fribbens *et al*., 2016). These results have further emphasized the potential for the determination of *ESR1* mutations to guide treatment decision making in ER‐positive MBC (Angus *et al*., 2017).

Another mechanism that potentially contributes to acquired endocrine therapy resistance is the occurrence of *ESR1* mRNA splice variants. *ESR1* splice variants have been described as having various effects on the transcriptional activity of the ER (Taylor *et al*., 2010), and are heterogeneously expressed in primary breast cancers (Poola and Speirs, 2001). The ERα∆5 splice variant is of particular interest, as preclinical experiments have reported that this variant exerts constitutional transcriptional activity (Bollig and Miksicek, 2000; Fuqua *et al*., 1991). However, to date, the putative role of *ESR1* splice variants with regard to endocrine resistance in MBC has not been assessed.

*ESR1* mutations and mRNA splice variants are likely to emerge during treatment and can therefore only be observed in tumor cells obtained during or after treatment. Thus, these investigations require metastatic tumor tissue obtained through biopsies, which can be technically challenging, or even impossible.

Circulating tumor cells (CTCs) and circulating tumor DNA (ctDNA) are alternative and minimally invasive means of assessing the characteristics of metastatic cancer cells. Theoretically, each acts as a different substrate for DNA, with DNA from CTCs coming from intact cancer cells, and ctDNA [which is part of the total cell‐free DNA (cfDNA)] is thought to originate mainly from apoptotic tumor cells (Haber and Velculescu, 2014). The introduction of very sensitive digital polymerase chain reaction (dPCR) assays has opened new avenues to determine the presence of mutations in ctDNA and in CTC‐derived DNA of patients with cancer. Although promising results have been achieved with the detection of *ESR1* mutations in cfDNA using dPCR (Chu *et al*., 2015; Fribbens *et al*., 2016; Guttery *et al*., 2015; Schiavon *et al*., 2015; Takeshita *et al*., 2015, 2016; Wang *et al*., 2016), the important advantage of using CTCs over cfDNA is that multiple parameters in multiple dimensions (DNA, RNA, and protein) can be measured in the same sample and can be associated with, for example, endocrine resistance. This implies that besides assessing mutations in CTC‐derived DNA, the characterization of RNA from CTCs permits the assessment of splice variants.

The current study set out to evaluate *ESR1* mutations and splice variants in CellSearch‐enriched CTCs of MBC patients before the start of first‐line endocrine therapy, and during progression under any line of endocrine therapy. The main objective was to determine whether these putative mechanisms for endocrine resistance are enriched in patients progressing on endocrine therapy. To this end, a cohort of MBC patients before the beginning of first‐line endocrine therapy for MBC was defined, as well as a cohort of MBC patients progressing under any line of endocrine therapy. Additionally, in a subgroup of these patients, the *ESR1* mutation status in CTCs was compared with patient‐matched cfDNA.

## 2 Materials and methods

### 2.1 Patients and treatment

The patients evaluated in this study were selected from two CTC studies comprising patients receiving endocrine therapy (study 06‐248 (Mostert *et al*., 2015; Onstenk *et al*., 2015b; Sieuwerts *et al*., 2011) and study 09‐405 (Reijm *et al*., 2016)). Six centers in the Netherlands and Belgium participated in these studies from February 2008 through March 2015. The patients were included in these studies if they had MBC, and a new line of endocrine therapy was begun. Blood was sampled before the start of endocrine therapy and/or at the time of progression to palliative endocrine treatment. At both of these time points, 10 mL of blood was drawn for CTC enumeration, and another 10 mL of blood was drawn for CTC characterization. In each participating center, the institutional board approved the study protocols (Erasmus MC ID MEC‐06‐248 and MEC‐09‐405). All patients provided written informed consent.

Two cohorts of patients were defined for the current study: a cohort starting first‐line endocrine therapy for MBC and a separate cohort progressing under any line of palliative endocrine therapy. Further eligibility criteria required that the patient had ≥5 CTCs/7.5 mL of blood at the time of the blood draw, to allow for the characterization of CTCs.

### 2.2 Enumeration and isolation of DNA and RNA from CTCs and cfDNA and ESR1 mutation determination

Details regarding the CTC enumeration and isolation of DNA/RNA from CTCs have been reported previously (Mostert *et al*., 2015; Onstenk *et al*., 2015b; Reijm *et al*., 2016; Sieuwerts *et al*., 2011). Briefly, in each patient, 10 mL of blood was drawn in CellSave tubes (Janssen Diagnostics, Raritan, NJ, USA) for CTC enumeration, which was performed on 7.5 mL of blood within 96 h of the blood draw using the CellSearch system (Janssen Diagnostics). Another 10 mL of blood was drawn into EDTA tubes for CTC characterization, and CTCs were isolated from 7.5 mL of blood within 24 h using the CellSearch system with the CellSearch profile kit (Janssen Diagnostics) (Fig. S1). Subsequently, DNA and RNA were isolated from enriched CTCs using the AllPrep DNA/RNA Micro Kit (Qiagen, Germantown, MD, USA) (Sieuwerts *et al*., 2011). For cfDNA analyses, the remainder of the EDTA blood (maximum of 2.5 mL) was centrifuged to isolate plasma within 24 h after the blood draw. Cell‐free DNA (cfDNA) was isolated from a total of 200 μL of plasma using the QIAamp circulating nucleic acid kit (Qiagen).

DNA from the CellSearch‐enriched CTC fractions and cfDNA from plasma were quantified using the Quant‐iT PicoGreen dsDNA Assay Kit (Thermo Fisher Scientific, Waltham, MA, USA). The DNA (0.1–1 ng·μL^−1^) was subjected to an *ESR1* target‐specific amplification of 15 cycles with TaqMan PreAmp Master Mix (Thermo Fisher Scientific), as recommended by the manufacturer, using the *ESR1* PreAmp primer combination (Table S1) at a final concentration of 400 nm each. The resulting pre‐amplified 136 base pair product covering the positions of all four *ESR1* hotspot mutation sites (D538G and Y537S/C/N) was diluted 10‐fold, and quantified via regular quantitative PCR (qPCR) for wild‐type (WT) *ESR1* using the same primers. The resulting Cq value was used to control the number of WT copies to be loaded onto the chips for dPCR analyses. The variant allele frequencies (VAF) of the studied mutations for *ESR1* were evaluated with mutation‐specific TaqMan assays (the primer and probe sequences are given in Table S1, and the reproducibility of these assessments in Fig. S2) via chip‐based dPCR (QuantStudio 3D; Thermo Fisher Scientific) according to the manufacturer's instructions. Positive and negative control DNA was always included in each dPCR run, and all of the analyzed DNA samples (CTC and cfDNA) were evaluated in duplicate.

Digital PCR was performed for four *ESR1* hotspot mutation sites (D538G and Y537S/C/N). Ten healthy blood donors were used to specify the cutoffs for the presence of *ESR1* mutations in CellSearch‐enriched samples. Seven of them had sufficient plasma available, and these samples were used to specify the cutoffs for the presence of *ESR1* mutations in cfDNA. The cutoff for the positivity for each individual assay was set at the highest VAF in the healthy blood donors plus 2.58 standard deviations (SD) (99% confidence interval) (Figs S3 and S4). The cutoffs were as follows: D538G = 0.6% (CTCs) and 1.0% (cfDNA), Y537S = 0.3% (for both CTCs and cfDNA), Y537N = 0.3% (CTCs) and 1.65% (cfDNA), Y537C = 0.5% (CTCs) and 0.65% (cfDNA). Both of the duplicate *ESR1* mutation measurements had to be above the cutoffs for a sample to be considered positive for a specific *ESR1* mutation.

### 2.3 Short tandem repeat analysis on patient‐matched CTC‐DNA and cfDNA

In a subset of samples with ≥ 10 CTCs and a high enough DNA content (≥ 30 ng) for which not all CellSearch‐enriched DNA was used for *ESR1* mutation analysis, a short tandem repeat (STR) analysis was performed to confirm that the CellSearch‐enriched DNA and cfDNA were indeed from the same donor. The PowerPlex 16 System (Promega, Madison, WI, USA), in combination with an ABI PRISM 3130xl Genetic Analyzer (Thermo Fisher Scientific) and genemarker v1.91 software (Softgenetics LLC, State College, PA, USA), was used to genotype the DNA, as recommended by the manufacturer's instructions.

### 2.4 ESR1 splice variants and expression in RNA from enriched CTCs

The measured ‘splice variant gene panel’ consisted of full‐length (FL) *ESR1* and *ESR1* splice variants ∆5, ∆7, 36 kDa, and 46 kDa. In addition, reference genes and epithelial genes were evaluated. Two microlitre of complementary DNA was pre‐amplified in 15 cycles with TaqMan assays and TaqMan PreAmp Master Mix (Thermo Fisher Scientific), as recommended by the manufacturer, using the gene panel combination given in Table S1. After pre‐amplification, each gene was individually measured via qPCR with the same TaqMan assay used in the pre‐amplification. Positive and negative controls were included in each individual experiment to monitor the reproducibility of the measurements (for reproducibility, see also Fig. S5).

The splice variants were assessed in CellSearch‐enriched fractions of 10 healthy blood donors to evaluate the possible leukocyte expression of FL *ESR1* and splice variants. The splice variant gene panel was always evaluated in duplicate, and the averages of the duplicate measurements were used for further calculation. Only those samples with sufficient mRNA signal (reference genes average ∆Cq<26.5) and epithelial signal (*KRT19/EPCAM* average ∆Cq<26.5), as described previously (Onstenk *et al*., 2015a; Sieuwerts *et al*., 2009, 2011), were used for further evaluation of splice variants. The ∆Cq values for the splice variants were calculated relative to the FL *ESR1*. In those cases where no expression could be measured for both the splice variant and the FL *ESR1*, the sample was excluded from the analysis.

### 2.5 Statistical considerations

The primary objective of this research was to investigate whether *ESR1* mutations were more frequently observed in CTCs of MBC patients progressing on endocrine therapy, than in those patients starting first‐line endocrine therapy. Based on data from the literature (Robinson *et al*., 2013; Toy *et al*., 2013), it was hypothesized that *ESR1* mutations in CTCs would be detectable in 30% of MBC patients experiencing progressive disease (PD) during palliative endocrine therapy and that *ESR1* mutations in CTCs would be present in 5% of those patients beginning palliative first‐line endocrine therapy. In order to detect this difference (α = 0.05 and β = 0.2), 44 MBC patients progressing on palliative endocrine therapy and 44 MBC control patients initiating first‐line endocrine therapy were needed.

Secondary objectives included (a) an assessment of *ESR1* mutations in cfDNA samples, and a comparison between the detection of *ESR1* mutations in cfDNA versus CTC; (b) an exploration of whether *ESR1* mutations measured in cfDNA are enriched under endocrine therapy; (c) an exploration of whether *ESR1* splice variants are more prevalent in those patients experiencing PD than in patients beginning first‐line endocrine therapy for MBC; and (d) an exploration of whether certain clinical factors are associated with the presence of *ESR1* mutations and/or splice variants.

Differences in the prevalence of *ESR1* mutation and splice variants between the baseline cohort and the progressing cohort were calculated using Fisher's exact test (two‐sided), while those patients with matched samples in the baseline and the progressing cohort were excluded from this analysis. Correlations were tested using Kendall's tau correlation coefficient, and the differences in splice variant ∆Cq values between groups were tested using the Kruskal–Wallis test. All of the analyses were performed using Stata/SE version 12 (StataCorp LP, College Station, TX, USA), and all of the data obtained from this study are available in Doc. S1.

## 3 Results

### 3.1 Patient characteristics

For the baseline cohort, a total of 43 patient samples were included, while the progressing cohort contained a total of 40 patient samples (Table**** 1). Most of the patients in the baseline cohort were not treated with any adjuvant chemotherapy (79%); however, 17 patients (40%) had been treated with adjuvant endocrine therapy. Samples in the progressing cohort originated mainly from patients progressing on first‐line (55%) or second‐line (30%) palliative endocrine therapy. Prior to the PD sample, 37 patients (93%) had received at least one line of AI treatment. Most patients (81%) in the baseline cohort experienced PD on endocrine therapy during the time of follow‐up. For six of these patients, matched samples from the baseline cohort and progressing cohort were available; however, for the other 29 patients, no PD sample was available, mainly because it was not collected. The median CTC count was higher in the baseline cohort (81 CTCs/7.5 mL) than in the progressing cohort (21 CTCs/7.5 mL).

**Table 1:** Baseline characteristics

Parameter	Description	Baseline cohort (*n* = 43)	PD cohort (*n* = 40)
Age at sample draw	Median age (range)	72 (37–83)	63 (35–88)
Adjuvant endocrine therapy (%)	No	26 (60)	26 (65)
	Yes, tamoxifen only	10 (23)	9 (23)
	Yes, tamoxifen + AI	5 (12)	4 (10)
	Yes, AI only	2 (5)	1 (2)
Adjuvant chemotherapy (%)	No	34 (79)	28 (70)
	Yes	9 (21)	12 (30)
Neoadjuvant therapies (%)	No	43 (100)	40 (100)
Number of previous lines endocrine therapy lines for MBC (%)	0	43 (100)	
	1		22 (55)
	2		12 (30)
	≥3		6 (15)
Endocrine therapy after start (BL cohort) or before PD (PD cohort) (%)	AI	30 (70)	25 (63)
	Tamoxifen	13 (30)	7 (17)
	Fulvestrant		8 (20)
Previous endocrine therapy lines for MBC (in case of inclusion at PD on ≥second‐line endocrine therapy) (%)	Yes, AI only		9 (23)
	Yes, AI + tamoxifen		6 (15)
	Yes, tamoxifen only		3 (7)
Progression on the current line (%)	Yes	35 (81)	40 (100)
CTC count	Median count (range)	81 (6–32492)	21 (5–2837)

### 3.2 ESR1 mutations in CTCs and matched cfDNA

In the six matched samples from the baseline and progressing cohorts, no *ESR1* mutations were detected. *ESR1* mutations were observed in the CTCs of two (5%) baseline cohort samples (2× Y537N) and three (8%) progressing cohort samples (2× D538G, 1× Y537S) (*P *=* *0.66) (Table**** 2). One of the patients in the baseline cohort with an *ESR1* mutation had received prior adjuvant treatment with tamoxifen, while the other patient had not received any prior adjuvant therapy. Two of the *ESR1* mutations in CTCs from patients in the progressing cohort, occurring after palliative first‐line therapy, were observed in one patient who had been treated with an AI and in one patient who had been treated with tamoxifen. The third *ESR1* mutation was observed in a patient progressing on fulvestrant as second‐line palliative endocrine therapy, who had received an AI as her first‐line treatment.

**Table 2:** Observed *ESR1* mutations in CTC and cfDNA samples. All patients in whom a mutation was called in either CTCs or cfDNA, along with clinical information. Shown percentages are variant allele frequencies. Called mutations are depicted in boldface

CTC code	Baseline CTCs	Baseline cfDNA	Adjuvant therapy	PD CTCs	PD cfDNA	Progression on therapy	Prior therapies for MBC
CTC798^a^	D538G (0.14%)	**D538G (1.93%)**	None	Not available	Not available		
CTC1581	Y537S (0.39%)^b^	**Y537S (0.47%)**	None	Not available	Not available		
	**Y537N (0.42%)**	Y537N (0.05%)					
CTC1571	**Y537N (3.77%)**	Not available	Tamoxifen	Not available	Not available		
CTC1007^a^	Not available	Not available	None	Y537S (0.01%)	**Y537S (9.26%)**	Fulvestrant	AI
CTC1364^a^	Not available	Not available	None	D538G (0.25%)	**D538G (40.05%)**	Tamoxifen	AI
CTC1565^a^	Not available	Not available	Tamoxifen + AI	D538G (0.14%)	**D538G (5.14%)**	Fulvestrant	AI
CTC1569	Not available	Not available	None	Y537N (0.25%)	**Y537N (1.96%)**	AI	
CTC1352	Not available	Not available	None	D538G (0.47%)	**D538G (20.93%)**	AI	Tamoxifen
CTC1567	Not available	Not available	None	**Y537S (1.98%)**	**Y537S (1.21%)**	Tamoxifen	
CTC1360	Not available	Not available	None	D538G (0.52%)	**D538G (2.86%)**	AI	
CTC1587	Not available	Not available	Tamoxifen	**D538G (0.84%)**	**D538G (15.98%)**	Fulvestrant	AI
CTC1406	Not available	Not available	Tamoxifen	**D538G (1.13%)**	**D538G (10.18%)**	AI	
CTC1393	Not available	Not available	None	D538G (0.18%) Y537C (0.23%)	**D538G (27.1%)Y537C (12.96%)**	AI	
CTC1410	Not available	Not available	Tamoxifen	D538G (0.37%)	**D538G (23.84%)**	AI	

^a^ STR analysis confirmed that the CTC‐DNA and cfDNA samples were from the same patient. For other samples, not enough DNA available for STR analysis. ^b^Average VAF positive, but negative in duplicate analysis.

Matched cfDNA and CTCs from the same time point were available from a subset of the patients in the baseline cohort (*n* = 18) and the progressing cohort (*n* = 26) (Table S2). Two *ESR1* mutations (1× D538G and 1× Y537S) (11%) were observed in cfDNA of the baseline cohort, and 12 *ESR1* mutations were observed in 11 patients (42%) in cfDNA of the progressing cohort (8× D538G, 2× Y537S, 1× Y537N, 1× Y537C) (*P *=* *0.04) (Table**** 2). In the four matched cfDNA samples from the baseline and progressing cohorts, no *ESR1* mutations were detected. Neither of the mutations found in cfDNA from the baseline cohort were observed in the CTCs (Table**** 2). In one of these patients, however, an Y537N mutation was observed in CTCs, but not in cfDNA. Neither of the patients with *ESR1* mutations in cfDNA from the baseline cohort had received any adjuvant therapy.

When the mutations in cfDNA from the progressing cohort samples were compared with the mutation status of the CTCs, three of three mutations observed in CTCs were confirmed in cfDNA. With one exception, variant allele frequencies (VAFs) of the mutations were much higher in cfDNA than in CTCs (Table**** 2). In addition, nine mutations in eight patients were observed in the cfDNA, but not in the CTCs. The mutations found in cfDNA of the progressing cohort occurred after first‐line endocrine therapies (*n* = 6), namely AIs (*n* = 5) and tamoxifen (*n* = 1), and after second‐line endocrine therapies (*n* = 5), namely fulvestrant (*n* = 3) and tamoxifen (*n* = 2). All of these latter patients had received an AI as first‐line palliative endocrine treatment.

From four patients with matched CTC‐cfDNA samples and discordant CTC versus cfDNA *ESR1* mutation results, unamplified DNA was available to perform STR analyses (Table**** 2). These analyses showed that both of the DNA fractions originated from the same patient, and thus excluded sample swapping.

### 3.3 ESR1 splice variants in CTCs

In order to assess the presence of *ESR1* splice variants in CTCs, RNA was extracted from CellSearch‐enriched CTCs and analyzed for the expression of four *ESR1* splice variants relative to full‐length *ESR1*. In the baseline cohort, 10 (23%) of the 43 samples were excluded from further analysis, because of insufficient quality of mRNA (*n* = 4) or lack of an epithelial signal (*n* = 6). In the progressing cohort, 17 (43%) of 40 samples had to be excluded because of insufficient quality of the mRNA (*n* = 2), lack of an epithelial signal (*n* = 6), or unavailable RNA (*n* = 9).

**Figure 1:**
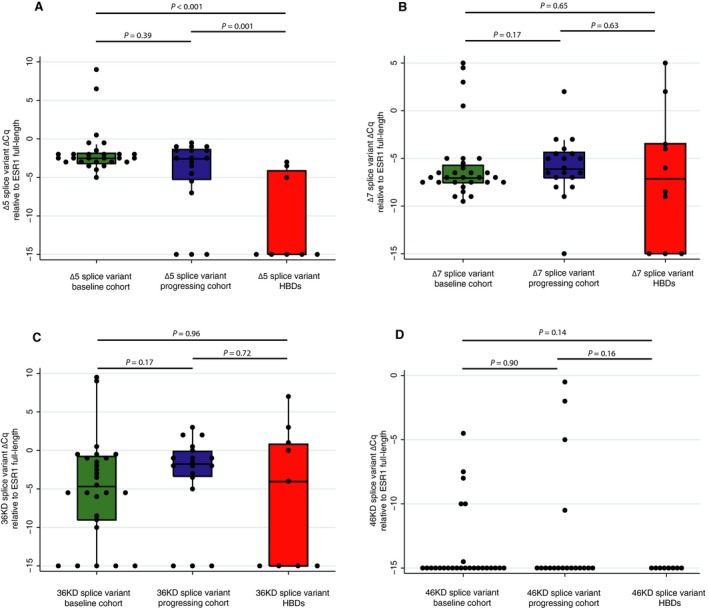
Occurrence of splice variants in the baseline cohort, the progressing cohort, and healthy blood donors (HBDs). Boxes demonstrate median and IQR; lines represent adjacent values (1.5*IQR). Observations were binned at ∆Cq of 0.5.

*ESR1* splice variant ∆Cq values relative to full‐length *ESR1* were not correlated with CTC counts (Fig. S6). ∆Cq values of the ∆5 splice variant relative to full‐length *ESR1* were significantly higher in patients than in healthy blood donors (HBDs) (Fig. 1A), but the ∆5 splice variant was not enriched in the progressing cohort, when compared to the baseline cohort (*P *=* *0.39). When four matched samples, taken from the baseline and progressing cohorts, were analyzed from patients receiving first‐line AI treatment, the ∆5 splice variant was enriched at PD in two of the patients (Fig. S7). The ∆7 and 36 kDa splice variants were similarly expressed in patient samples and HBDs (Fig. 1B,C). Nevertheless, for the four matched samples from the baseline and progressing cohorts, the ∆7 and 36‐kDa splice variants were enriched at PD in one and three patients, respectively (Fig. S7). The 46‐kDa splice variant was only observed in patient samples and not in HBDs; however, this did not reach statistical significance (Fig. 1D).

## 4 Discussion

The current study evaluated whether *ESR1* mutations and splice variants were enriched in CTCs from MBC patients progressing under endocrine therapy. No enrichment of any of these putative resistance mechanisms in CTCs was observed after endocrine therapy. However, cfDNA analyses did reveal an enrichment of *ESR1* mutations at the time of progression on endocrine therapy, when compared with before the initiation of first‐line endocrine treatment.

The observation that *ESR1* mutations were more frequently observed in cfDNA than in CTCs suggests that cfDNA is a more sensitive substrate for the analysis of *ESR1* mutations than CTCs enriched by the FDA‐approved CellSearch system. This is also reflected by the VAFs in the CTCs, which were generally low (range: up to 3.8%), as opposed to the VAFs in the cfDNA, which were generally much higher (range: up to 40%). One explanation for this difference could be the presence of contaminating leukocytes following the CellSearch enrichment of CTCs, which we had previously reported to be around 1000 leukocytes (Sieuwerts *et al*., 2009), thereby decreasing the sensitivity for the detection of *ESR1* mutations in CTCs. However, our experiments suggesting those amounts of leukocytes after CellSearch profile were conducted in healthy donors in perfect circumstances with quick processing. For the current study, materials from patients were used which were sometimes shipped from distant sites and processed within 24 h, which may have resulted in a higher number of contaminating leukocytes. Therefore, the numbers of leukocytes that are present after CellSearch enrichment may be even higher than 1000 leukocytes in some samples, which is likely to decrease sensitivity for detecting *ESR1* mutations even more in those samples. Although cfDNA analysis is also challenged by the contamination of wild‐type DNA, our results suggest that this is less of an issue in cfDNA than in CTCs.

The stringency of the cutoffs for *ESR1* mutations, now arbitrarily set at the highest VAF observed in HBDs plus 2.58xSD (representing the 99% confidence interval), could have played a role in the limited sensitivity of *ESR1* mutation detection in CTCs. When less stringent cutoffs based on the highest VAF in HBDs were explored (data not shown), slightly more *ESR1* mutations were observed in CTCs; however, the majority of these mutations were not observed in cfDNA, suggesting that relaxing the cutoffs for *ESR1* mutation positivity may lead to false‐positive findings. This stresses the need to include HBDs, and to be stringent with setting the cutoff value for *ESR1* mutation positivity. Interestingly, the current study observed one *ESR1* mutation exclusively present in CTCs, but not in cfDNA. This finding suggests that some *ESR1* mutations may be missed by cfDNA analysis only, albeit this observation may be merely anecdotal.

The current study is among the first to assess *ESR1* mutations in a cohort of patients beginning first‐line endocrine treatment for MBC. While it has already been recognized that primary breast cancers rarely harbor *ESR1* mutations (Jeselsohn *et al*., 2014; Toy *et al*., 2013), most studies thus far have evaluated patients who had been pretreated with palliative endocrine therapy, suggesting that these mutations become enriched during treatment with AIs (Schiavon *et al*., 2015). Here, it has been confirmed that *ESR1* mutations are not frequently present in MBC patients before first‐line endocrine therapy, and are enriched in MBC patients progressing under endocrine therapy.

Most of the patients in this study having an *ESR1* mutation progressed on AI treatment or had previously been treated with an AI. In three of the patients, *ESR1* mutations were observed after progression on fulvestrant, suggesting that although it has been reported that fulvestrant is more effective than AIs in *ESR1*‐mutant patients (Fribbens *et al*., 2016; Spoerke *et al*., 2016), mutant subclones can still be observed at PD on fulvestrant therapy. Of further note is the fact that in the current study the observed mutations in the baseline cohort occurred in those patients who were not pretreated with AIs, or who received no pretreatment with endocrine therapy at all. In addition, an *ESR1* mutation was observed in CTCs and cfDNA of one patient progressing on first‐line palliative tamoxifen therapy, but who had not received any AI treatment, also not in the adjuvant setting. These findings are in line with the observations of multiple groups (Guttery *et al*., 2015; Jeselsohn *et al*., 2014; Takeshita *et al*., 2015), who reported *ESR1* mutations in metastatic biopsies or cfDNA of patients who had only received tamoxifen, or no pretreatment at all. This could also fit with the observations by Wang *et al*. (2016), who reported that *ESR1* mutations were sometimes present in primary breast cancers of patients at extremely low VAFs.

In the current study, the *ESR1* splice variant ∆5 was expressed at higher levels in the CellSearch‐enriched samples from MBC patients than in HBD samples; however, we found no enrichment of this splice variant during endocrine therapy for MBC. The ∆7, 36‐kDa, and 46‐kDa splice variants were not significantly more highly expressed in patients versus HBDs. The fact that full‐length *ESR1* and splice variants were also measured in a subset of HBDs suggests that leukocytes, which are known to express *ESR1* (Scariano *et al*., 2008), may also express these splice variants. This clearly complicates the analysis of *ESR1* splice variants measured in CellSearch‐enriched CTC fractions, where one thousand‐fold of leukocytes is still present. In metastatic prostate cancer, the presence of the androgen receptor (AR) splice variant V7 in CTCs was previously demonstrated to be strongly associated with resistance to endocrine agents (Antonarakis *et al*., 2014), but not to chemotherapy (Antonarakis *et al*., 2015; Onstenk *et al*., 2015a; Scher *et al*., 2016). It should, however, be noted that splice variants of *ESR1* in breast cancer differ importantly from splice variants of the AR, as *ESR1* splice variants are also expressed in healthy breast tissue (Poola and Speirs, 2001), and full‐length AR and splice variants are typically absent in CellSearch‐enriched fractions of HBDs (Onstenk *et al*., 2015a). It should also be kept in mind that, in the current study, only a limited number of samples could be evaluated for the presence of splice variants. However, given that the *ESR1* splice variant ∆5 has been linked to endocrine resistance (Bollig and Miksicek, 2000; Fuqua *et al*., 1991), is CTC‐specific expressed, and that we found anecdotal evidence of enrichment of this splice variant in paired samples, further research of this splice variant in CTCs is warranted.

## 5 Conclusion

*ESR1* mutations and splice variants in CellSearch‐enriched CTCs were not enriched in MBC patients progressing on palliative endocrine therapy, but *ESR1* mutations were enriched in those patients when they were assessed in cfDNA. Therefore, cfDNA appears to be a more sensitive and robust source for detecting *ESR1* mutations than DNA from CellSearch‐enriched CTCs. However, the use of other CTC enrichment methods might yield better results (Denis *et al*., 2016). To improve the sensitivity and specificity of detecting mutations and splice variants, and to really exploit the potential power of CTCs, characterization of pure CTCs with single cell isolation systems is probably required (Swennenhuis and Terstappen, 2015). Until that has been proven feasible and superior to analysis of cfDNA, the detection of *ESR1* mutations in cfDNA rather than CTCs is recommended. The increased incidence of *ESR1* mutations in cfDNA at the time of progression on endocrine therapy further adds to the evidence that emergence of *ESR1* mutations is involved in resistance to endocrine therapy in MBC.

## Data Accessibility

**Dataset S1.** Overview of all data from this study.

## Author contributions

NB, AMS, MPJ, JAF, JWM, and SS designed the study; NB, AS, NMV, WO, SRV, and MD performed the laboratory experiments. JK, MPJ, and JWM supervised the experiments; LYD, PH, FEJ, AJ, and CMS included patients in the clinical study for this analysis; NB, WO, and AB collected the clinical data; NB analyzed the data and compiled statistics; NB and AMS wrote the manuscript, which was reviewed, edited, and approved by all authors.

## Supporting information


**Fig. S1.** Flowchart of study procedures.
**Fig. S2.** Reproducibility of *ESR1* mutation measurements in CTCs and cfDNA.
**Fig. S3.** Cut‐offs for *ESR1* mutations in CTCs.
**Fig. S4.** Cut‐offs for *ESR1* mutations in cfDNA.
**Fig. S5.** Reproducibility of splice variant measurements in T47D cell line.
**Fig. S6.** Correlation between *ESR1* splice variant delta Cq values and CTC counts.
**Fig. S7.** Dynamics of splice variants in 4 matched samples at baseline and PD.
**Table S1.** Primer and probe sequences.
**Table S2.** Spike‐in experiments with and without pre‐amplification.
**Table S3.** Characteristics of patients in cfDNA subgroup analysis.
