# Supplementary material for: Estrogen receptor mutations and splice variants determined in liquid biopsies from metastatic breast cancer patients
Source: Mol Oncol. 2017 Nov 17;12(1):48–57. doi: 10.1002/1878-0261.12147 (PMC5748489; doi:10.1002/1878-0261.12147)
Supplement: Supplementary file 1 — Fig. S1. Flowchart of study procedures. Fig. S2. Reproducibility of ESR1 mutation measurements in CTCs and cfDNA. Fig. S3. Cut‐offs for ESR1 mutations in CTCs. Fig. S4. Cut‐offs for ESR1 mutations in cfDNA. Fig. S5. Reproducibility of splice variant measurements in T47D cell line. Fig. S6. Correlation between ESR1 splice variant delta Cq values and CTC counts. Fig. S7. Dynamics of splice variants in 4 matched samples at baseline and PD. Table S1. Primer and probe sequences. Table S2. Spike‐in experiments with and without pre‐amplification. Table S3. Characteristics of patients in cfDNA subgroup analysis. [file MOL2-12-48-s001.pdf]

Supplemental Table 1: Primer and probe sequences used for RT-qPCR and digital PCR

Description	Assay	Symbol	Forward primer	Reverse primer	FAM Taqman probe*	VIC Taqman probe*	bp product
Reference gene	qPCR	*GUSB*			Hs99999908_m1		81
Reference gene	qPCR	*HMBS*			Hs00609297_m1		64
Leukocyte control marke[illegible]	qPCR	*PTPRC*			Hs00236304_m1		81
Epithelial control marke[illegible]	qPCR	*KRT19*			Hs01051611_gH		66
ESR1 wild type	qPCR	*ESR1-WT*			Hs00174860_m1		62
Epithelial control marke[illegible]	qPCR	*EPCAM*	AGTTTGCGGACTGCACTTCA	AATACTCGTGATAAATTTTGGAT[illegible]	AAGGAGATCACAACGCGT		72
ESR1 splice variant	qPCR	*ESR1_DELTA-*[illegible]	GAGCTGGTTCACATGATC	CATGTCGAAGATCTCCAC	TTTCCCTGGTTCCTGGCAC		225
ESR1 splice variant	qPCR	*ESR1_DELTA-*[illegible]	TTTGTGTGCCTCAAATCTA	TTCATGCTGTACAGATGC	CCATGCCTTTGTTACAGAATTAAGC		254
ESR1 splice variant	qPCR	*ESR1-36KD*	CCAAGAATGTTCAACCACAAC[illegible]	GCACGGTTCATTAACATCTTTCT[illegible]	TATTTATGTTCCAGTCCCACCTGAGTAGCAAAGTG		143
ESR1 splice variant	qPCR	*ESR1-46KD*	CATTCTCCGGGACTGCGGTA	GTACTGGCCAATCTTTCTCTGCC	TGAGGCCAAATTCAGATAATCGACGCCAGGG		140
ESR1 PreAmp	dPCR	*ESR1 PreAmp*	AGGCATGGAGCATCTGTACA	TTGGTCCGTCTCCTCCA			136
ESR1 mutation	dPCR	*ESR1_D538G*	CAGCATGAAGTGCAAGAACGT	TGGGCGTCCAGCATCTC	CCCTCTATGGCCTGCT	CCCCTCTATGACCTGCT	63
ESR1 mutation	dPCR	*ESR1_Y537S*	CAGCATGAAGTGCAAGAACGT	TGGGCGTCCAGCATCTC	CCCTCTCTGACCTGC	CCCCTCTATGACCTGC	63
ESR1 mutation	dPCR	*ESR1_Y537C*	CAGCATGAAGTGCAAGAACGT	TGGGCGTCCAGCATCTC	CCCCTCTGTGACCTG	TGCCCCTCTATGACCTG	63
ESR1 mutation	dPCR	*ESR1_Y537N*	CTGTACAGCATGAAGTGCAAG[illegible]	TGGGCGTCCAGCATCTC	TGCCCCTCAATGAC	TGGTGCCCCTCTATGAC	68
ESR1 mutation	dPCR	*ESR1_Y537C* [1]	AGGCATGGAGCATCTGTACA	TTGGTCCGTCTCCTCCA	TGCCCCTCTGTGACCTG[illegible]	TGGTGCCCCTCTATGAC[illegible]	136
ESR1 mutation	dPCR	*ESR1_Y537N* [1]	AGGCATGGAGCATCTGTACA	TTGGTCCGTCTCCTCCA	TGCCCCTCAATGACCTG[illegible]	TGGTGCCCCTCTATGAC[illegible]	136
Reference; synthetic *ESR1* _Y537C[1]	dPCR	GTCTTCCCACCTACAGTAACAAAGGCATGGAGCATCTGTACAGCATGAAGTGCAAGAACGTGGTGCCCCTCTGTGACCTGCTGCTGGAG ATGCTGGACGCCCACCGCCTACATGCGCCCACTAGCCGTGGAGGGGCATCCGTGGAGGAGACGGACCAAAGCCACTTGGCCACTGCG GGCTC					181
Reference; synthetic *ESR1* _Y537N[1]	dPCR	ATGCTGGACGCCCACCGCCTACATGCGCCCACTAGCCGTGGAGGGGCATCCGTGGAGGAGACGGACCAAAGCCACTTGGCCACTGCG GGCTC					181
Reference; synthetic *ESR1* _Y537S[1]	dPCR	ATGCTGGACGCCCACCGCCTACATGCGCCCACTAGCCGTGGAGGGGCATCCGTGGAGGAGACGGACCAAAGCCACTTGGCCACTGCG GGCTC					181

* MGB NFQ probe; Hs assays concern validated intron-spanning FAM labeled Taqman gene expression assays from ThermoFisher Scientific

Schiavon G, Hrebien S, Garcia-Murillas I, Cutts RJ, Pearson A, Tarazona N, et al. Analysis of ESR1 mutation in circulating tumor DNA demonstrates evolution during therapy for metastatic breast cancer. Sci Transl Med. 2015 Nov

Supplementary Table 2. Various amounts of plasma input of a healthy blood donor and the resulting VAF with and without spiking of D538G, and with and without pre-amplification.

mL plasma start	D538G spiked-in	without pre-amplification			with pre-amplification		
		pg in dPCR*	%VAF	*%SD*	pg in dPCR*	%VAF	*%SD*
**4.0**	yes	11909	18.4%	*0.7%*	9248	16.1%	*0.3%*
**2.0**	yes	7869	14.6%	*3.1%*	7245	17.4%	*0.1%*
**1.0**	yes	4648	20.2%	*7.8%*	8817	15.3%	*0.1%*
**0.5**	yes	2243	28.3%	*5.5%*	7389	15.7%	*0.1%*
**0.2**	yes	2798	17.6%	*4.2%*	6510	25.8%	*1.1%*
**2.0**	no	*1866*	*0.0%*	*0.0%*	12142	0.5%	*0.1%*
**1.0**	no	*<200*	*0.0%*	*0.0%*	8066	0.2%	*0.0%*
**0.2**	no	*386*	*0.0%*	*0.0%*	11929	0.2%	*0.0%*
**tissue**	no	116	14.8%		1722	15.9%	*1.0%*

* < 2,000 pg DNA input in the dPCR is considered a too low input for a reliable analysis of low mutated samples

Supplementary Table 3. Characteristics of patients in cfDNA subgroup analysis

Parameter	Description	Baseline cohort (n=18)	Progressing cohort (n=26)
Age at sample draw	Median age (range)	68 (46 - 83)	64 (35 - 88)
Adjuvant endocrine therapy			
	No	11 (61%)	18 (69%)
	Yes, tamoxifen only	5 (28%)	4 (15%)
	Yes, tamoxifen + AI	2 (11%)	3 (12%)
	Yes, AI only		1 (4%)
Adjuvant chemotherapy			
	No	15 (83%)	18 (69%)
	Yes	3 (17%)	8 (31%)
Number of previous lines endocrine therapy lines for MBC			
	0	18 (100%)	
	1		16 (62%)
	2		8 (31%)
	≥3		2 (7%)
Endocrine therapy after start (baseline cohort) or before PD (progressing cohort)			
	AI	11 (61%)	16 (62%)
	Tamoxifen	7 (39%)	3 (11%)
	Fulvestrant		7 (27%)
Previous endocrine therapy lines for MBC (in case of inclusion at PD on ≥2nd-line endocrine therapy)			
	Yes, AI only		6 (23%)
	Yes, AI + tamoxifen		2 (8%)
	Yes, tamoxifen only		2 (8%)
Progression on the current line	Yes	13 (77%)	26 (100%)

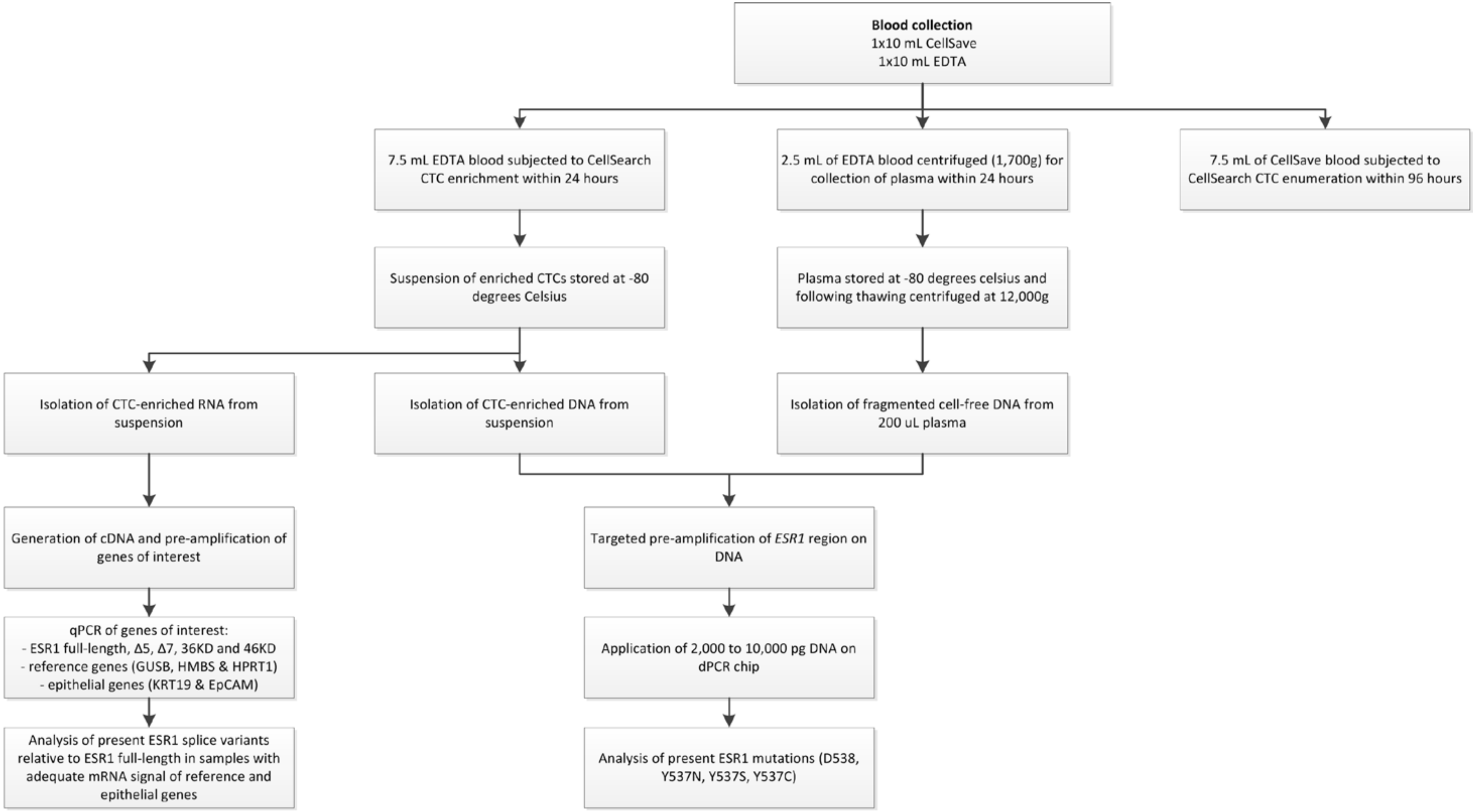

Supplemental Figure 1. Flowchart of study procedures.

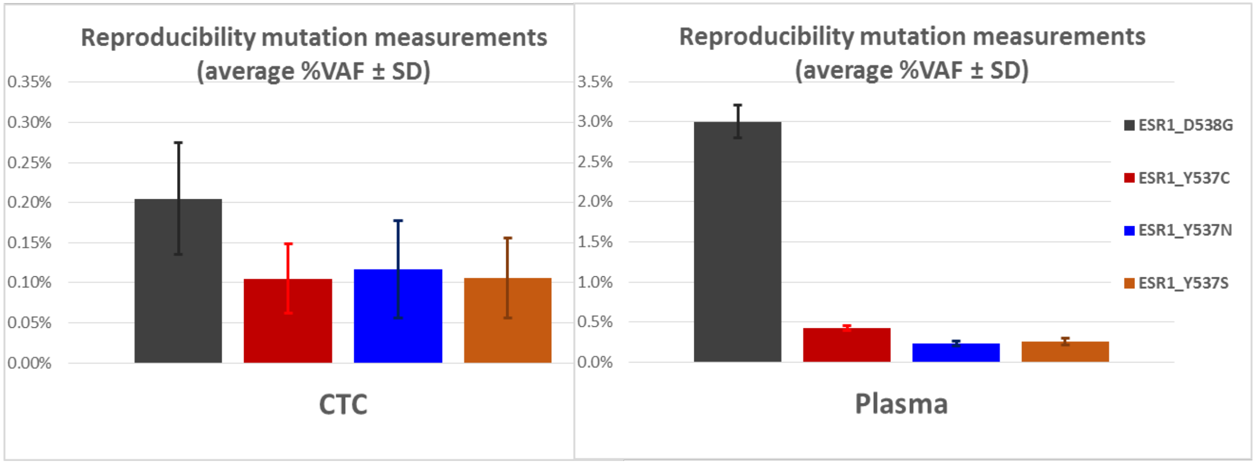

Supplemental Figure 2. Reproducibility of *ESR1* mutation measurements in CTCs and cfDNA. Boxes demonstrate average VAF along with standard deviations (SDs) based on duplicate measurements.

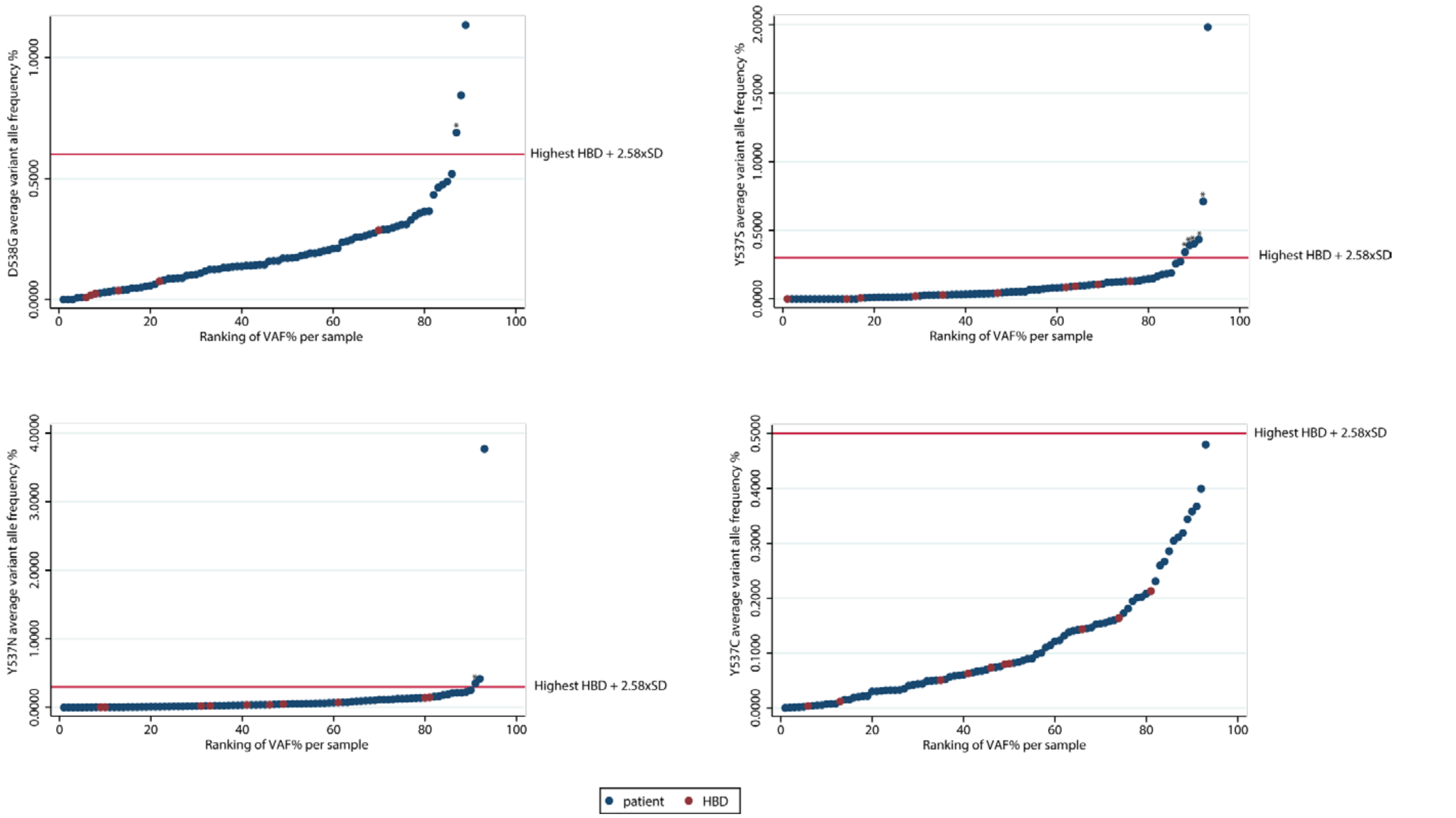

Supplemental Figure 3. Cut-offs for *ESR1* mutations in CTCs. Each point represent one sample. Samples were ranked according to the VAF and cut-offs were set at the VAF in the highest HBD (depicted with red dot) + 2.58xSD. Stars indicate that the although the average VAF% was above the cut-off, duplicate experiments were not both above the cut-off.

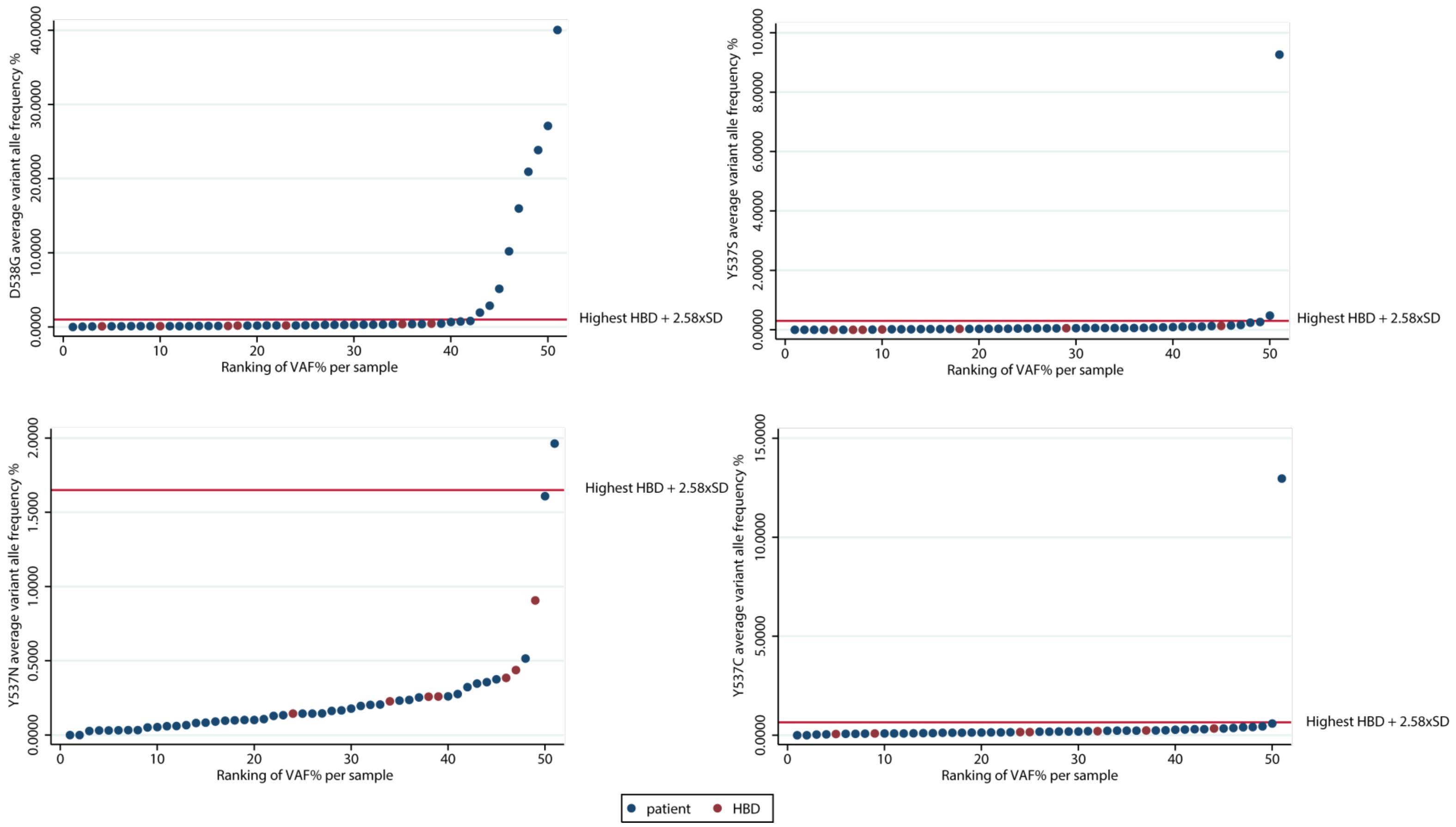

Supplemental Figure 4. Cut-offs for *ESR1* mutations in cfDNA. Each point represent one sample. Samples were ranked according to the VAF and cut-offs were set at the VAF in the highest HBD (depicted with red dot) + 2.58xSD.

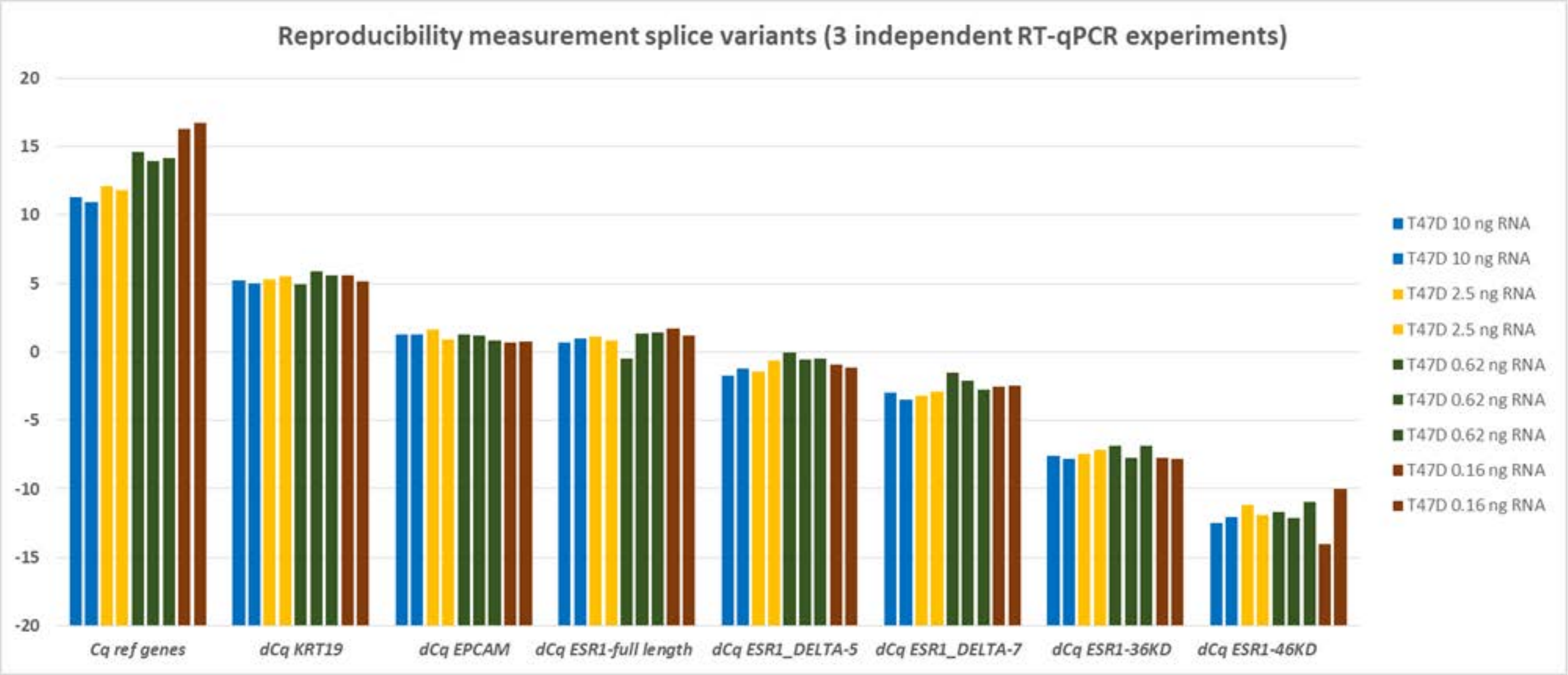

Supplemental Figure 5. Reproducibility of splice variant measurements in T47D cell line with various inputs of RNA. Various inputs (10 ng, 2.5 ng, 0.62 ng and 0.16 ng) of RNA from cell line T47D were measured in the splice variant panel. Bars represent Cq values of reference genes for all inputs, and ΔCq values (corrected for reference genes) for cytokeratin 19 (KRT19), EpCAM, *ESR1*-wildtype/full length and *ESR1* splice variants.

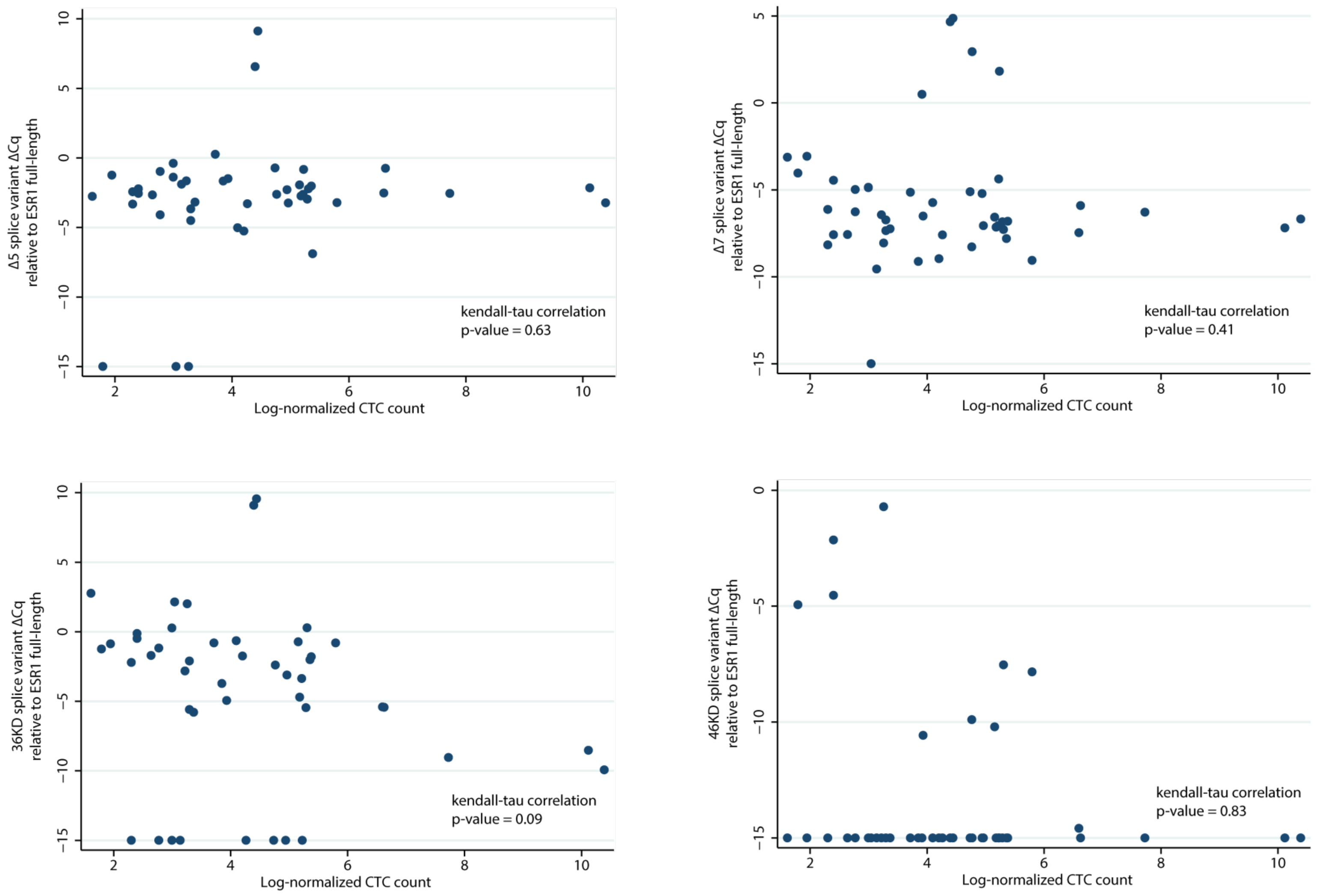

Supplemental Figure 6. Correlation between *ESR1* splice variant ΔCq values (relative to *ESR1* full-length) and CTC counts. CTC counts were log-normalized to compress the figure. Samples in which both the *ESR1* splice variant and *ESR1* full-length had no Cq were excluded. Samples in which only *ESR1* full-length but no *ESR1* splice variant was measured got a ΔCq values (relative to *ESR1* full-length) of -15.

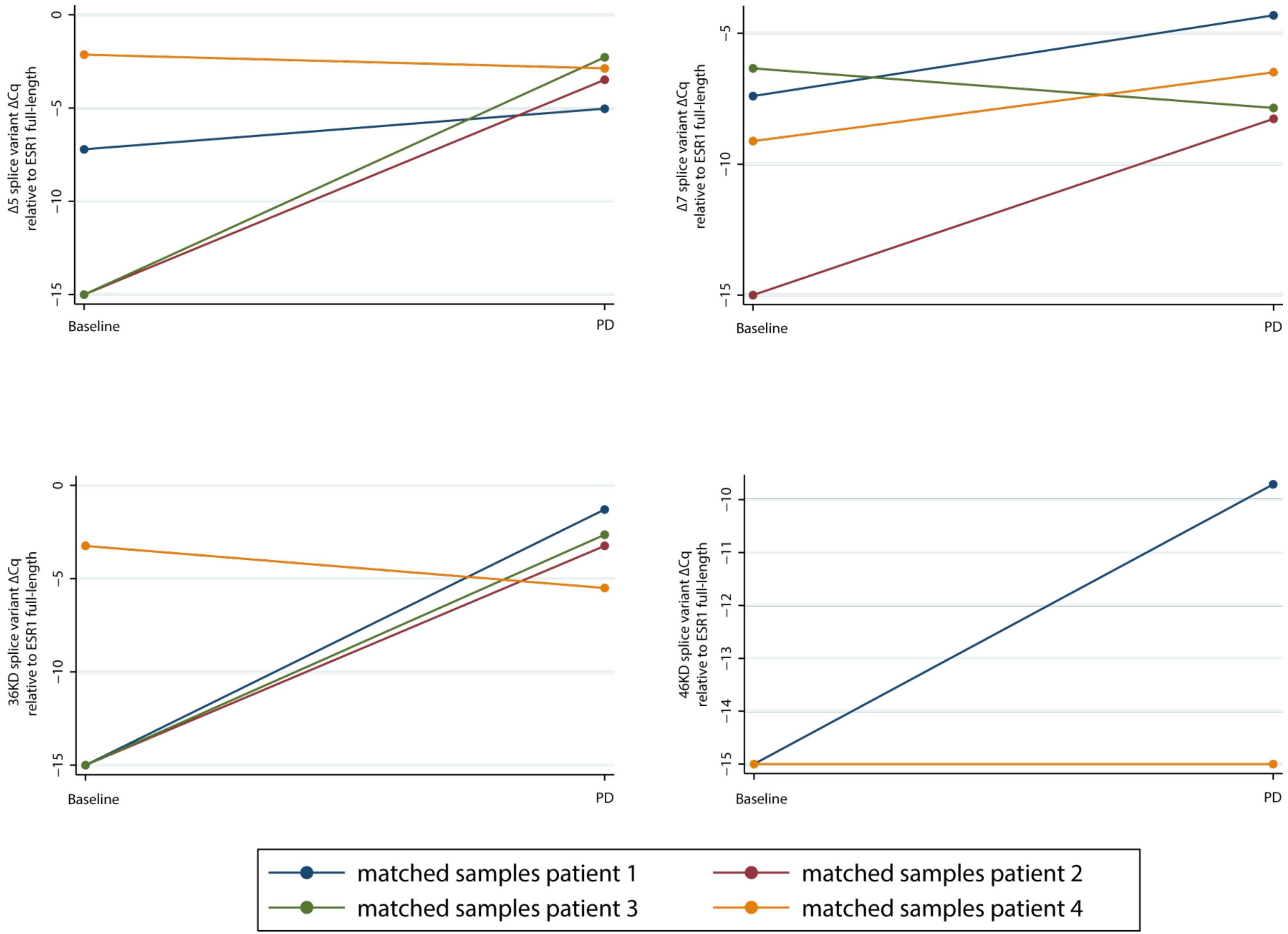

Supplementary Figure 7. Dynamics of splice variants in 4 matched samples at baseline and PD. All patients received first-line AI treatment.
